# Antidepressants affect gut microbiota and *Ruminococcus flavefaciens* is able to abolish their effects on depressive-like behavior

**DOI:** 10.1038/s41398-019-0466-x

**Published:** 2019-04-09

**Authors:** Iva Lukić, Dmitriy Getselter, Oren Ziv, Oded Oron, Eli Reuveni, Omry Koren, Evan Elliott

**Affiliations:** 1 Molecular and Behavioral Neuroscience, The Azrieli Faculty of Medicine, Bar-Ilan University, Henrietta Szold St. 8, Safed, Israel; 2 Microbiome Research, The Azrieli Faculty of Medicine, Bar-Ilan University, Henrietta Szold St. 8, Safed, Israel; 3 Drug discovery Laboratories, The Azrieli Faculty of Medicine, Bar-Ilan University, Henrietta Szold St. 8, Safed, Israel

## Abstract

Accumulating evidence demonstrates that the gut microbiota affects brain function and behavior, including depressive behavior. Antidepressants are the main drugs used for treatment of depression. We hypothesized that antidepressant treatment could modify gut microbiota which can partially mediate their antidepressant effects. Mice were chronically treated with one of five antidepressants (fluoxetine, escitalopram, venlafaxine, duloxetine or desipramine), and gut microbiota was analyzed, using 16s rRNA gene sequencing. After characterization of differences in the microbiota, chosen bacterial species were supplemented to vehicle and antidepressant-treated mice, and depressive-like behavior was assessed to determine bacterial effects. RNA-seq analysis was performed to determine effects of bacterial treatment in the brain. Antidepressants reduced richness and increased beta diversity of gut bacteria, compared to controls. At the genus level, antidepressants reduced abundances of *Ruminococcus*, *Adlercreutzia*, and an unclassified Alphaproteobacteria. To examine implications of the dysregulated bacteria, we chose one of antidepressants (duloxetine) and investigated if its antidepressive effects can be attenuated by simultaneous treatment with *Ruminococcus flavefaciens* or *Adlercreutzia equolifaciens*. Supplementation with *R. flavefaciens* diminished duloxetine-induced decrease in depressive-like behavior, while *A. equolifaciens* had no such effect. *R. flavefaciens* treatment induced changes in cortical gene expression, up-regulating genes involved in mitochondrial oxidative phosphorylation, while down-regulating genes involved in neuronal plasticity. Our results demonstrate that various types of antidepressants alter gut microbiota composition, and further implicate a role for *R. flavefaciens* in alleviating depressive-like behavior. Moreover, *R. flavefaciens* affects gene networks in the brain, suggesting a mechanism for microbial regulation of antidepressant treatment efficiency.

## Introduction

During the past decade, there has been an increase in understanding of how the gut microbiota affects various aspects of brain development and function, as well as behavior. For example, studies on germ free mice revealed that gut bacteria influence development of stress response, appropriate maturation and function of microglia, affect anxiety, social and depressive-like behaviors, along with alterations in gene expression and neurochemistry of different brain regions^1–5^. Regarding the etiology of depression, it has been shown that germ free mice exhibit less behavioral despair, along with higher brain serotonin levels, in comparison to their conventional counterparts^2,6^. The importance of gut bacteria in development of mood disorders was further confirmed by several recent studies showing that patients with depression had altered diversity and composition of gut microbiota, and these changes were causally related to depressive-like behavior in rodent models^7,8^.

Antidepressants are major drugs used for treatment of depression^9,10^. Some of the most effective antidepressants act as inhibitors of serotonin and/or norepinephrine reuptake which leads to increased synaptic concentrations of these neurotransmitters^11–14^. However, even though they are in use for >50 years, the precise molecular mechanisms of their therapeutic action are still not completely understood. Of particular importance, it is not clear what is the biological mechanism behind the variability of efficacy of antidepressants between different individuals. It is presumed that their therapeutic effects are achieved through slow onset auto-receptor down-regulation, and subsequent adaptation of downstream neural signaling pathways, including promotion of neural plasticity^15–18^. Besides this, although the antidepressants are considered to be efficient, still the relative risk reduction of relapse by the continuous treatments was estimated to be 50–60%^19,20^. All that point out further need for better understanding of antidepressant actions, along with searching for new treatments, or complementary ways to improve the efficiency of current antidepressant medication.

Considering the evidence for a role of microbiota in depressive behavior, we hypothesize that antidepressants also change gut microbiota composition, and through modulation of the microbiota, at least partly, exert their antidepressant effects. Indeed, some antidepressants were shown to have antimicrobial effects *in vitro* against several groups of microorganisms, and inhibit number of processes in microorganisms, such as slime production and bacterial motility^21–23^. On the other hand, serotonin and noradrenaline, which are found in high amounts in the gut, can promote growth and virulence in certain bacteria, acting as interkingdom signaling molecules^24–27^. Also, recently it was found that knockout of rat serotonin transporter disrupted gut bacteria homeostasis, augmentating early life stress effects as well^28^. Considering that there is considerable variation in the microbiome between individuals^29^, we may also consider that microbiome variation may be partly a mechanism for the variability of antidepressant efficacy among different individuals.

In addition to the effects of antidepressants on microbiota, microbiota may also effect depressive-like behaviors through modulation of neurotransmitters and other key molecules. Microbiota can produce neuroactive compounds, including neurotransmitters, that may influence host physiology and behavior^30,31^. In addition, host microbiota can influence the production of serotonin by enterochromaffin cells in the host gut^32^. This is interesting, considering that the gut is the main source of serotonin. This can provide a further mechanism to support the hypothesis that antidepressants may partially mediate their effects through regulation of microbiota.

To explore the aforementioned hypothesis, we treated BALB/c mice with one of five antidepressants, commonly used in clinical practice and different in their mode of action. The choice of BALB/c strain for the study was based on their natural characteristics of exhibiting higher depressive-like behavior^33–37^ and anxiety^36,38,39^ compared to other strains. Furthermore, they were shown to be responsive to chronic antidepressant treatments that reduce their immobility in test of behavioral despair^37,40,41^. All these, make BALB/c strain as a suitable model to study antidepressant responses relevant for depressive disorder. Indeed, our results demonstrated that antidepressants change diversity and composition of gut bacterial communities and one of the identified bacterial species, affected by their treatment, is able to mediate alleviation of depressive behavior.

## Methods and materials

### Animals

Male BALB/c OlaHsd mice, purchased from Harlan (Israel), were used in the study. The mice were housed under reverse 12 h light/dark cycle conditions, with water and food available ad libitum. All animals were group housed with 3–5 animals per cage. When studying antidepressant effects on microbiota, animals were divided into 3 cages, in order to minimize cage effect. Animals that developed illnesses during the experiments were excluded from the study. The experiments started when mice were 8–10 weeks of age. All experimental protocols were approved by the Animal Care and Use Committee of Bar-Ilan University. Animals were randomly assigned to each experimental group.

### Antidepressant and bacterial treatments

**Fig. 1:**
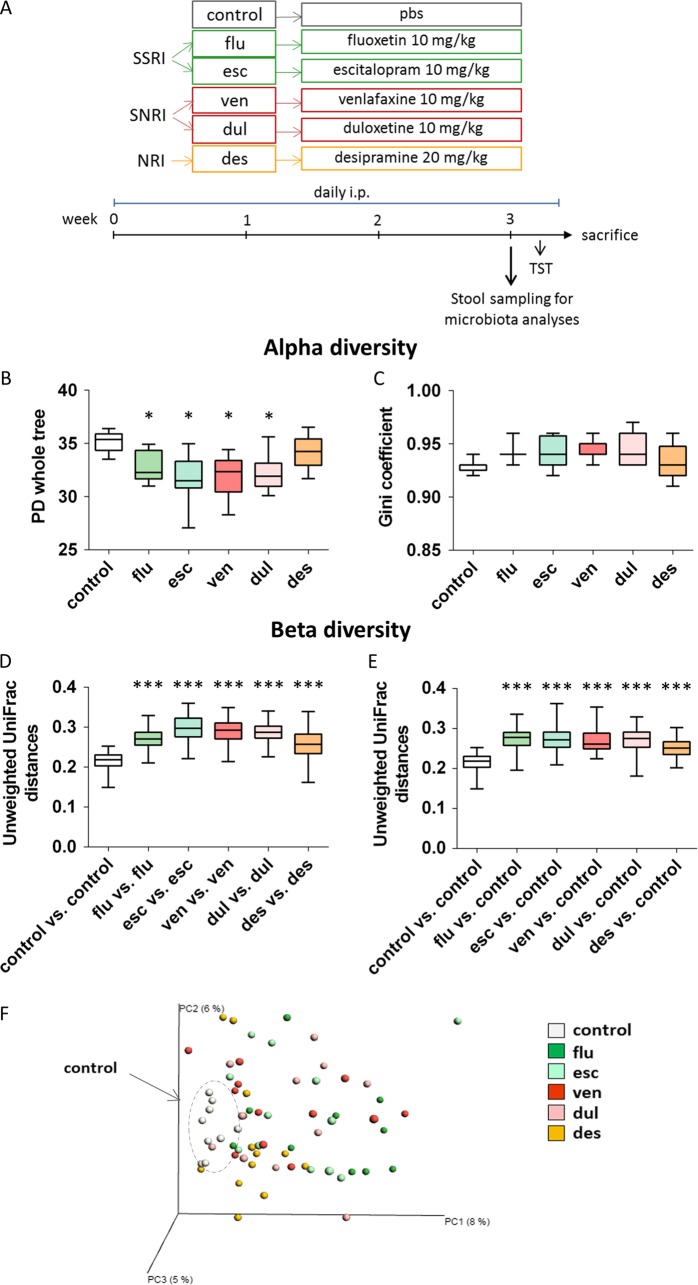
Antidepressants alter diversity of gut microbiota. **a** Experimental design of the study examining antidepressant effects on gut microbiota. **b**, **c** Measures of alpha diversity. All antidepressants, except desipramine, reduced richness of microbial communities (PD whole tree is shown) (**b**), but there was no changes in the evenness (Gini coeficient is shown) (**c**). **d**–**f** Evaluations of beta diversity. Bacterial communities of mice treated with antidepressants displayed higher unweighted UniFrac distances in comparison with controls (**d**) and microbial communities of control group were more similar to each other than to the communities of antidepressant treated mice (**e**). Unweighted UniFrac-based principal coordinates analysis (PCoA) plot used to visualize microbial communities of all antidepressant treated and control mice (the percentage of variation explained by the principal coordinates is indicated on the axes) (**f**). * *p* < 0.05, ** *p* < 0.01, FDR corrected, nonparametric t-tests with 999 Monte Carlo permutations in comparison to control group; *n* = 9 (control), *n* = 11 (flu), *n* = 12 (esc), *n* = 12 (ven), *n* = 11 (dul), *n* = 12 (des), animals per group; data represent mean ± SEM. *flu* fluoxetine, *esc* escitalopram, *ven* venlafaxine, *dull* duloxetine, *des* desipramine

**Fig. 2:**
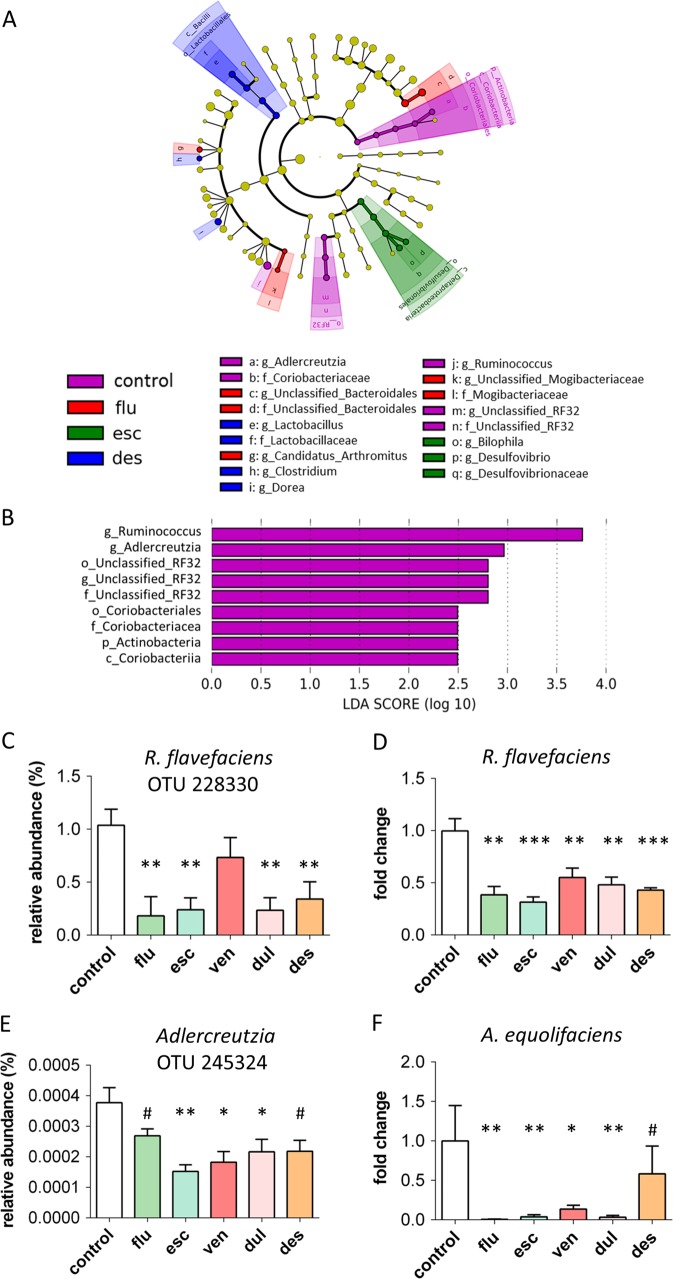
Bacterial taxa changed by antidepressants. **a**, **b** Results of LEfSe analyses. Taxonomic cladograms representing bacterial taxa differently abundant in stool samples from different groups: VIOLET- bacterial taxa more abundant in control group, compared to all antidepressant treated groups; RED - bacterial taxa more abundant in fluoxetine treated mice; GREEN - bacterial taxa more abundant in escitalopram treated mice; BLUE - bacterial taxa more abundant in desipramine treated mice. There were no bacterial taxa that were more abundant in venlafaxine or duloxetine treated mice compared to all other groups (**a**). Visualization of bacterial taxa, ranked by effect size, that were more abundant in control group compared to all antidepressant treated groups (p < 0.05, LDA>2) (**b**). **c**–**f** Validation of *Ruminococcus* and *Adlercreutzia* levels. Reduced relative abundance of OTU 228330, assigned to species *Ruminococcus flavefaciens* (**c**) and reduced levels of *Ruminococcus flavefaciens* quantified by qRT-PCR (**d**) in stool samples of antidepressant treated mice compared to controls. Reduced relative abundance of OTU 245324, with 98% of similarity to *Adlercreutzia equolifaciens* (**e**), and reduced levels of *Adlercreutzia equolifaciens* quantified by qRT-PCR, in stool samples of antidepressant treated mice compared to controls (**f**). Levels of gut bacteria were normalized to control group. ^#^ 0.1 > *p* > 0.05, *p < 0.05, **p < 0.01, ***p < 0.001, Mann–Whitney tests in comparison to control group, followed by FDR correction; n = 9 (control), *n* = 11 (flu), *n* = 12 (esc), *n* = 12 (ven), *n* = 11 (dul), *n* = 12 (des), animals per group; data represent mean ± SEM. *flu* fluoxetine, *esc* escitalopram, *ven* venlafaxine, *dul* duloxetine, *des* desipramine

All five antidepressants used in the study were purchased from Sigma-Aldrich. Antidepressants were diluted in PBS, in the following doses: fluoxetine 10 mg/kg, escitalopram 10 mg/kg, venlafaxine 10 mg/kg, duloxetine 10 mg/kg and desipramine 20 mg/kg, and delivered by daily i.p. in volume of 8 ml/kg (Fig. 1a). In all experiments, mice were treated with antidepressants for 21 days before stool collection or beginning of behavioral tests. The effective doses were selected according to the available literature regarding their therapeutic concentrations in mice^40–44^. The control group received corresponding volume of PBS. In the initial experiment (Figs. 1 and 2), the number of animals used per experimental group were n = 9 (control), *n* = 11 (flu), *n* = 12 (esc), *n* = 12 (ven), *n* = 11 (dul), *n* = 12 (des).

When effects of both antidepressant and bacteria were studied, the antidepressant was prepared and delivered as previously described.

**Fig. 3:**
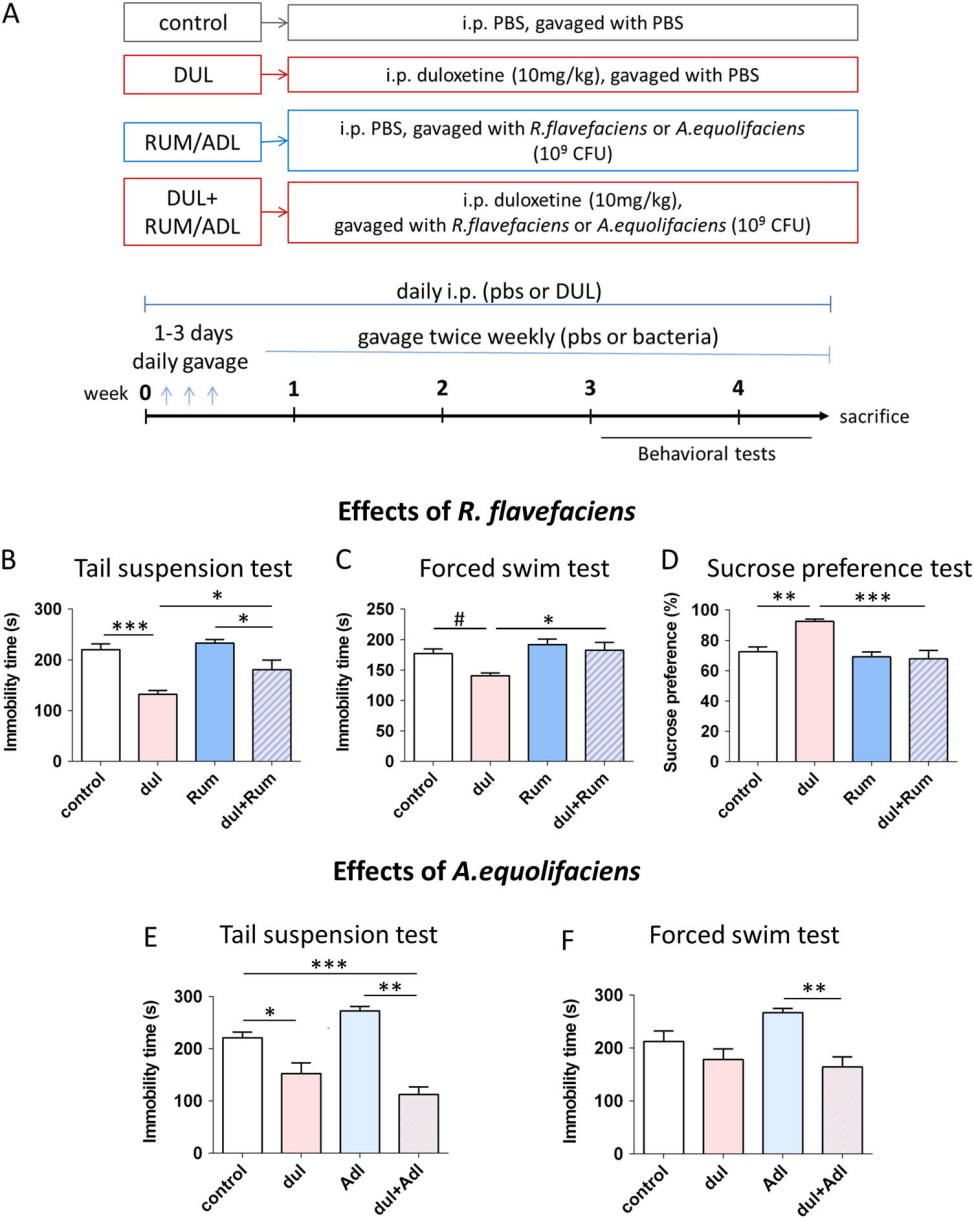
*R. flavefaciens*, but not *A. equolifaciens*, abolished antidepressive effect of duloxetine. **a** Experimental design of studies examining behavioral effects of duloxetine and bacterial treatments (*R. flavefaciens* or *A. equolifaciens*). Mice were chronically treated with antidepressant and/or bacteria, followed by behavioral testing. **b**–**d** Effects of duloxetine and *R. flavefaciens* on depressive-like behavior. *R. flavefaciens* supplementation abolished antidepressive effect of duloxetine in tail suspension test (**b**), forced swim test (**c**) and sucrose preference test (**d**). *n* = 10 (control), *n* = 10 (dul), *n* = 11 (Rum), *n* = 11 (dul+Rum), animals per group. **e**, **f** Effects of duloxetine and *A. equolifaciens* on depressive-like behavior. Duloxetine still reduced depressive-like behavior after *A. equolifaciens* treatment in tail suspension test (**e**) and forced swim test (**f**). *n* = 10 (control), *n* = 10 (dul), *n* = 11 (Adl), *n* = 10 (dul+Adl), animals per group. ^#^0.1 > *p* > 0.05, *p < 0.05, **p < 0.01, ***p < 0.001, Tukey’s post hoc test; data represent mean ± SEM. *dul* duloxetine, *Rum* *R. flavefaciens, Adl* *A. equolifaciens*

Bacterial species *Ruminococcus flavefaciens* 17 (DSM 25089) and *Adlercreutzia equolifaciens* FJC-B9 (DSM 19450) were obtained from DSMZ and cultured in appropriate mediums anaerobically^45,46^. On the day of bacterial treatment, per each mouse, 10^9^ colony-forming units (CFU) were suspended in 200 μl of sterile PBS and immediately delivered by gavage. Control groups were gavaged by the same volume of PBS. Mice were treated with bacteria daily for the first 3 days, and then twice weekly till the end of the experiment (Fig. 3a). Gavage was never performed on the day of behavioral experiment. In the experiment with treatment with *Ruminococcus*, the number of animals used per experimental group were *n* = 10 (control), *n* = 10 (dul), *n* = 11 (Rum), *n* = 11 (dul + Rum). In the experiment with treatment with *Adlercreutzia*, the number of animals used per experimental group were *n* = 10 (control), *n* = 10 (dul), *n* = 11 (Adl), *n* = 10 (dul+Adl).

### Behavioral testing

Depressive-like behavior of mice was assessed after 21^st^ day of treatment, using tail suspension test (TST), forced swim test (FST) and sucrose preference test (SPT) (for details see Supplement). Locomotor activity was assessed in a separate group of animals, after 21^st^ day of treatment as well, using open field test and rotarod (for details see Supplement). All tests were done during the dark phase, between 10 a.m. and 3 p.m., and animals were acclimated to the behavior room for 1 h before testing (except for sucrose preference test that was performed in home cages). Between each of behavioral tests, mice had at least one day of rest. On the test day, the i.p. injections of an antidepressant or pbs was done 1 h before testing.

### Stool collection, DNA extraction and sequencing of 16 s rRNA gene

Mice fecal samples were collected under aseptic conditions, 1 h after i.p. injection of antidepressants or PBS, and stored at −80 °C until further analyses. DNA was isolated using the PowerSoil DNA isolation kit (MoBio Laboratories) according to the manufacturer’s instructions following an initial 2 minute beadbeating step (BioSpec). (For details about 16s rRNA gene amplification see Supplement.)

### Bioinformatic analyses of 16S rRNA gene sequences

Obtained 16s rRNA gene sequencing data were analyzed by QIIME 1 pipeline^47^ (for details see Supplement). Alpha diversity (within community diversity) was estimated by Faith’s phylogenetic diversity (PD)^48^ and Chao1^49^, as a measure of community richness, and by Gini coefficient, as a measure of community evenness^50^. Beta diversity (between communities diversity) was calculated using unweighted and weighted UniFrac distances^51^. The diversity parameters were compared between groups using a nonparametric t-test with Monte Carlo permutations (999) to calculate *p* values, and Benjamini and Hochberg FDR method was used afterwards to correct *p* values for multiple comparisons between different pairs of groups.

Differences in relative abundances of bacterial taxa between groups were identified using the linear discriminant analysis (LDA) effect size (LEfSe) method (version 1.0)^52^ as well as permutational multivariate analysis of variance (PERMANOVA). LEfSe uses the nonparametric Kruskal–Wallis rank-sum test to detect features which have significantly different abundances between groups. Then, it performs LDA to estimate the effect size of each feature (alpha significance level was set at 0.05 and an effect-size threshold was set at 2).

### Bacterial detection by PCR

Bacterial abundances of the stool samples used in 16S rRNA gene sequencing were verified by quantitative real-time PCR (qRT-PCR). It was performed using Fast Start Universal SYBR Green Master (Rox) (Roche) and ViiA™7 Real-Time PCR System (Life Technologies). PCR consisted of 40 cycles, using melting temperature of 95 °C for ten seconds per cycle, and an annealing/elongation temperature of 60 °C or 57 °C, as appropriate, of thirty seconds per cycle. Relative quantification by ddCt method was used to verify bacterial abundances in the gut. The primer sequences used in the reactions are indicated in the Supplementary Table S1. In order to detect bacterial species in bacteria treated mice, DNA from stool samples were amplified by PCR as described above, and then ran on a 2% agarose gel to visualize PCR bands.

### Brain tissue isolation and RNA extraction

Mice were sacrificed by rapid decapitation and brains were quickly removed. The medial prefrontal cortices (mPFC) were isolated using brain matrix and gauge 13 (from the slice between 3 mm and 1 mm anterior to bregma), and immediately frozen on dry ice. Total mPFC RNA was extracted from six samples per experimental group using RNeasy Mini Kit (Qiagen) according to the manufacture protocol. NanoDrop 1000 (Thermo Scientific) and Qubit were used to determine the purity and concentration of RNA, respectively. Bioanalyzer 2100 (Aligent Technologies) was used to verify RNA integrity number (RIN), and all samples displayed RIN greater than 7.90.

### mRNA sequencing

From 100 ng of total RNA of each sample, mRNA enrichment was done by NEBNext Poly(A) mRNA Magnetic isolation Module (NEB # E7490), followed by preparation of RNA libraries using NEBNext Ultra RNA Prep kit (NEB #E7530), according to manufacturer’s protocols. Libraries’ concentrations were determined by Qubit, while quality and size distribution was analyzed using Bioanalyzer 2100. The sequencing was performed at the Technion Genome Center, Haifa with the Illumina HiSeq 2500. Fastq files are available at GEO under the accession number GSE129359.

### Bioinformatic analyses of mRNA sequencing

The quality of the sequenced data, as well as read length distributions after trimming, was evaluated by FASTQCT (0.11.5.). The reads were mapped to the Mus musculus reference genome, GRCm38.p5, using the Tophat2 software. (For details regarding differential expression analyses see Supplement). The weighted gene correlation network analysis (WGCNA) R software package was applied to the entire set of normalized gene counts with the aim of identifying gene modules affected by the treatments^53^ (for details see Supplement). Enrichment analyses for the Gene ontology (GO) terms (molecular function, biological process and cellular component) were performed using online ToppGene Suite software. GO terms were considered to be significant when the Benjamini and Hochberg FDR adjusted *p* value was below 0.05. For protein-protein interaction (PPI) network analysis, the STRING database followed by Cytoscape (version 3.2.1) network construction was used (for details see Supplement).

### Serotonin and noradrenalin brain levels

mPFC was extracted as previously described for RNA collection. After weighting, brain tissue was homogenized in 0.01 N HCl with 1 mM EDTA and 4 mM sodium metabisulfite on ice. Levels of serotonin and noradrenaline were measured using Serotonin Research ELISA and Noradrenaline Research ELISA kits (LDN, Nordhorn, DE), respectively, according to manufacturer’s protocols.

### Statistical analyses

Statistical analyses were done using SPSS. When the assumptions of normality and homogeneity of variances were met, the data were analyzed by ANOVA. When these assumptions were violated, non-parametric tests were used. Namely, the effects of antidepressants and bacteria on behavior and neurotransmitter brain levels were analyzed by one-way or two-way ANOVA, as appropriate. Comparison between groups was performed by Dunnett or Tukey post hoc test, as appropriate. The effects of antidepressants on bacterial levels in gut were analyzed by Kruskal–Wallis test, followed by pairwise Mann-Whitney tests. Level of significance was set at *p* < 0.05.

## Results

### Antidepressants affect gut microbiota composition

To investigate whether antidepressants may alter gut microbiota, we chose five different antidepressants common in clinical practice and different in their mode of action. We used two selective serotonin reuptake inhibitors (SSRIs) - fluoxetine and escitalopram, two serotonin norepinephrine reuptake inhibitors (SNRIs) - venlafaxine and duloxetine, and desipramine that acts as norepinephrine reuptake inhibitor. Since antidepressants require at least three weeks to show their therapeutic effects, we characterized the gut microbial community after 21 day of daily antidepressant treatment (Fig. 1a; OTU table with all detected bacteria can be found in Supplementary Table 1). Antidepressants were injected i.p. in order to provide a specific concentration of antidepressants directly to the gut. Treatment by drinking water would cause potential cofounders in the analysis of specific drug effects on microbiota, due to potential variability among experimental groups in drinking water volumes and differential kinetics of breakdown of antidepressants in drinking water. Nonetheless, one important limitation of our approach is that daily i.p. administration adds an element of stress to the experimental outline which can affect experimental findings.

Analyses of alpha diversity revealed that all antidepressants, except desipramine, reduced the richness of microbial communities (Fig. 1b; Supplemental Figure S1a, b), but did not affect their evenness (Fig. 1c). Beta diversity measures of gut microbiota were also affected by all studied antidepressants, but more pronounced effects were observed in unweighted UniFrac analyses (Fig. 1d–f) than in weighted UniFrac (Supplemental Figure S1c–e). Namely, beta diversity of fecal microbial communities from mice receiving antidepressants was higher than beta diversity of control samples (Fig. 1d, f). Further, microbial communities of control group were more similar to each other than when they were compared to samples of any of antidepressant treated groups (Fig. 1e, f).

In order to identify bacterial taxa which differed in relative abundances in antidepressant treated mice in comparison to controls, we analyzed the 16S rRNA gene sequencing results using linear discriminant analysis (LDA) effect size (LEfSe) algorithm. A comparison between control mice and all antidepressant groups together revealed that *Ruminococcus*, *Adlercreutzia* and an undefined genus in the order RF32, class Alphaproteobacteria were less abundant in antidepressant treated mice (Fig. 2a, b). When analyzing the effects of each individual antidepressant in comparison to control using pairwise comparisons, the same genera were shown to be less abundant in escitalopram, venlafaxine, duloxetine and desipramine groups, but not in fluoxetine group (Supplemental Figure S2). Further, in both the overall effects of antidepressants and in the pairwise analysis, the differences in genus *Adlercreutzia* contributed to observed decreased abundance of family *Coriobacteriaceae*, order Coriobacteriales, class Coriobacteriia, phylum Actinobacteria in antidepressant groups (Fig. 2a, b; Supplemental Figure S2). In addition to the LEfSe analysis, we further analyzed the 16S rRNA gene sequencing data with PERMANOVA (Supplementary Table 2). At the genus level, both *Ruminococcus* and *Adlercreutzia* were effected by antidepressant treatment (p < 0.05), although only Adlercreutzia remained significant after corrections for multiple comparisons. In direct pairwise analysis between control and each of the antidepressant groups, some antidepressants significantly decreased levels of *Ruminococcus*, although they were no longer significant after corrections for multiple comparisons. Therefore, the findings of decrease in *Ruminococcus* and *Adlercreutzia* were more significant in the LEfSe analysis, and we further explored these findings using qRT-PCR.

Validation of antidepressant-induced decrease in *Ruminococcus* and *Adlercreutzia* levels was done by qRT-PCR (undefined genus in order RF32 could not be analyzed by qRT-PCR because of the shortage of knowledge about its sequence). In order to determine which species of *Ruminococcus* was most affected, examination of the OTU table revealed that the abundance of the OTU assigned to *Ruminococcus flavefaciens* was altered by most of the antidepressant treatments (Kruskal–Wallis test H = 27.88, *p* < 0.001) (Fig. 2c). The qRT-PCR verified that *R. flavefaciens* was indeed less abundant in all antidepressant groups compared to controls (Kruskal–Wallis test H = 22.33, *p* < 0.001) (Fig. 2d). In the genus *Adlercreutzia*, *Adlercreutzia* *equolifaciens* is the only characterized species of this genus. The OTU that had the highest (98%) similarity to *Adlercreutzia equolifaciens* and was the most abundant in mice gut, was also decreased by most of antidepressants (Kruskal–Wallis test H = 16.06, *p* < 0.01) (Fig. 2e). Likewise, the qRT-PCR results showed that all antidepressants except desipramine, reduced levels of *A*. *equolifaciens* in the mice gut (Kruskal–Wallis test H = 26.60, *p* < 0.001) (Fig. 2f).

### *R. flavefaciens* but not *A*. *equolifaciens* reduces antidepressive effects of duloxetine

In our next group of experiments, we wanted to explore the hypothesis that *R. flavefaciens* or *A. equolifacien* may mediate the effects of antidepressant treatment on depressive-like behavior. With that aim, we treated BALB/c mice with antidepressant, bacteria (*R. flavefaciens* or *A. equolifacien*s), or both, and then performed behavioral testing (Fig. 3a). For this group of experiments, we chose antidepressant duloxetine because it decreased both *R. flavefaciens* and *A. equolifacien*s, as well as it showed the biggest effect in TST in BALB/c mice (Supplemental Figure S3). Presence of gavaged bacteria in the gut was confirmed by detecting *R. flavefaciens* or *A*. equolifaciens in stool samples of treated mice (Supplemental Figure S4).

First, we examined effects of *R. flavefaciens* in the TST. As expected, duloxetine induced a significant effect (two-way ANOVA: F_dul_ = 30.70, *p* < 0.001), and duloxetine treated animals displayed significantly less immobility, compared to control animals (Fig. 3b). Interestingly, there was also a significant effect of *R. flavefaciens* (two-way ANOVA: F_Rum_ = 5.90, *p* < 0.05), and animals treated with both *R. flavefaciens* and duloxetine displayed significantly more immobility compared to those treated with duloxetine alone (Fig. 3b). Therefore, *R. flavefaciens* treatment was able attenuate the duloxetine effect in the TST. In the FST, significant effects were also obtained by both duloxetine (two-way ANOVA: F_dul_ = 5.70, *p* < 0.05) and *R. flavefaciens* (two-way ANOVA: F_Rum_ = 8.74, *p* < 0.01) (Fig. 3c). Specifically, animals concomitantly treated with *R. flavefaciens* and duloxetine were more immobile than animals treated with duloxetine alone, and displayed behavior comparable to control animals (Fig. 3c). Therefore, *R. flavefaciens* treatment was able to abolish the effect of duloxetine treatment in the FST. The effects of *R. flavefaciens* in TST and FST were not confounded by locomotor deficits (Supplemental Figure S5a, b). Together, these results demonstrate that *R. flavefaciens* can attenuate duloxetine effects in behavior despair paradigms.

Further, we examined the effect of *R. flavefaciens* on anhedonia, by SPT. Both the antidepressant and the bacteria showed significant effects (two-way ANOVA: F_dul_ = 6.42, *p* < 0.05; F_Rum_ = 13.73, *p* = 0.001) (Fig. 3d). Namely, duloxetine increased mice preference for 2% sucrose, while it did not change the sucrose preference in the group receiving *R. flavefaciens* together with the drug (Fig. 3d). Also, sucrose preference was higher in group receiving duloxetine than in group receiving both duloxetine and *R. flavefaciens* (Fig. 3d). Moreover, the interaction of duloxetine and the bacteria treatment was significant in SPT (two-way ANOVA: F_dul*Rum_ = 8.34, *p* < 0.01) (Fig. 3d). In conclusion, *R. flavefaciens* supplementation reduced or abolished antidepressive properties of duloxetine.

In addition, the effect of *R. flavefaciens* on general gastrointestinal health was evaluated by number of fecal pellets in open field test (Supplemental Figure S5c). *R. flavefaciens* abolished constipation induced by duloxetine. The decreased defecation in a new environment induced by duloxetine can be attributed to its anxiolytic effect as well, yet we note that there were no changes in time spent in center of the open field arena by any of treatments, which is also taken as sign of anxiety behavior^54^ (data not shown).

Additionally, we determined whether antidepressant effects can be modulated by *A*. *equolifaciens*. As in the previous experiment, duloxetine had significant effects on immobility time in TST (two-way ANOVA: F_dul_ = 55.63, *p* < 0.001) and to lesser degree in FST (two-way ANOVA: F_dul_ = 6.52, *p* < 0.05) (Fig. 3e, f). However, when the drug was given together with *A*. *equolifaciens*, it still exhibited its antidepressant properties (Fig. 3e, f). Therefore, contrary to *R. flavefaciens*, *A*. *equolifaciens* did not abolish antidepressive effects of duloxetine.

### *R. flavefaciens* up-regulates mitochondrial genes while down-regulates neural genes in mPFC

In order to reveal mechanisms by which *R. flavefaciens* exerts its effects on the brain, we did whole transcriptome analyses on RNA extracted from the mPFC from each of the four experimental groups: control, duloxetine treated, *R. flavefaciens* treated, and those treated with both *R. flavefaciens* and duloxetine. The mPFC is known to be a significant center of behavior regulation, receiving inputs from different limbic structures, and has been implicated in both depression and antidepressant treatment effects^55,56^.

### Differential expression analyses

**Fig. 4:**
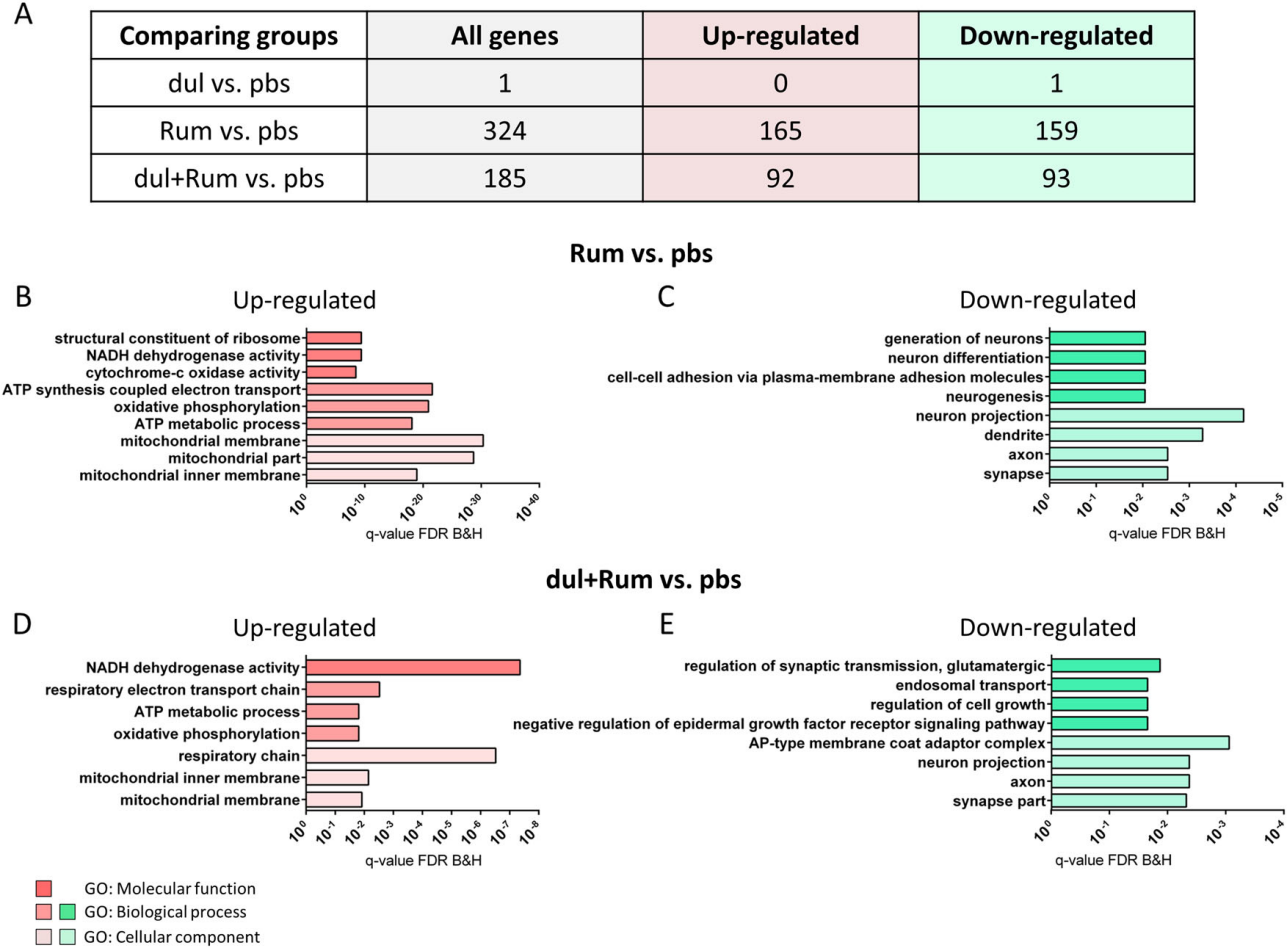
Differentially expressed genes (DEGs) after duloxetine and *R. flavefaciens* treatments. **a** The total number of DEGs, as well as the number of genes that are up-regulated and down-regulated by the particular treatment, are represented in the table. **b**, **c** Gene ontology (GO) enrichment analyses of genes up-regulated (**b**) and down-regulated (**c**) by *R. flavefaciens* treatment. **d**, **e** GO enrichment analyses of genes up-regulated (**d**) and down-regulated (E) by concomitant duloxetine and *R. flavefaciens* treatment. Bars representing GO terms show Benjamini and Hochberg FDR adjusted *p* values. *n* = 6 animals per experimental group. *dul* duloxetine, *Rum R. flavefaciens*

RNA-seq data were firstly analyzed by differential expression analyses (DEA) (Supplementary Table 3). Duloxetine treatment alone, compared to control, changed expression of only one gene, *Adrb1*, one of the norepinephrine receptors (Fig. 4a). In contrast*, R. flavefaciens* treatment resulted in 324 differentially expressed genes (DEGs), in comparison to controls (Fig. 4a). GO analyses revealed that genes up-regulated by *R. flavefaciens* treatment were enriched for mitochondrial processes (Fig. 4b), especially oxidative phosphorylation, while the down-regulated genes were enriched with genes involved in neural plasticity (Fig. 4c). When the group treated by both *R. flavefaciens* and duloxetine was compared to controls, there were 185 DEGs (Fig. 4a). Interestingly, concomitant *R. flavefaciens* and duloxetine treatments also resulted in the up-regulation of genes enriched for mitochondrial energy metabolism (Fig. 4d), and down-regulation of genes enriched for neural plasticity (Fig. 4e). When the group receiving both the bacteria and duloxetine was compared to the group receiving duloxetine alone, only one gene, *Dpysl2*, was shown to be differentially expressed, i.e., down-regulated. The list of all DEGs can be found in Supplemental Table 3. Therefore, treatment with *R. flavefaciens* induces expression changes of genes related to mitochondrial and neuronal process in the mPFC.

### Weighted gene correlation network analysis

In order to determine clusters of genes whose expression was associated with duloxetine and *R. flavefaciens* treatment, we analyzed our data using weighted gene correlation network analysis (WGCNA). Namely, DEA are useful method for independent discovery of genes, with high confidence, which expression levels are changed by a treatment. On the other hand, WGCNA approach enabled us to discover groups of genes, which individually may not have been identified by DEA, but whose changes in expression are strongly mutually correlated and related with the same direction to a treatment. Further, this method could be of greater relevance for understanding disturbed biological pathways.

**Fig. 5:**
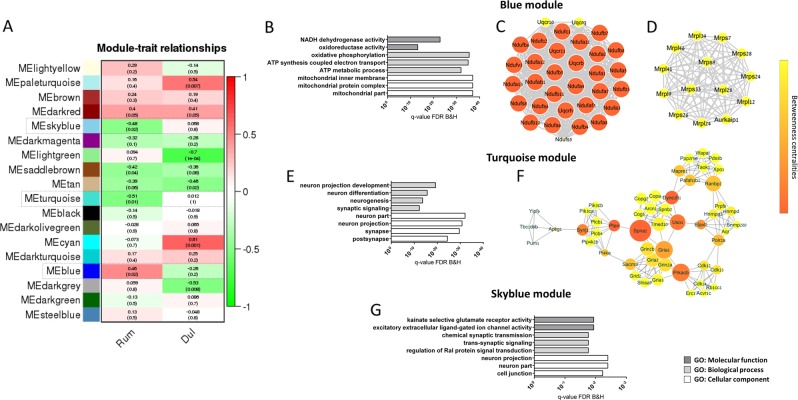
Results of weighted gene correlation network analysis (WGCNA). **a** Table of module-trait relationship reports Kendall’s correlation coefficients, and its corresponding *p* values, between the eigengene value of each module and the particular treatments. Modules related to *R. flavefaciens* treatment, and were not correlated with the same directionality with duloxetine treatment, are emphasized by dashed lines. **b**–**g** Gene ontology (GO) and protein-protein interaction (PPI) network analysis of WGCNA modules significantly related to *R. flavefaciens* treatment. GO enrichment analyses (**b**) and PPI network analysis (**c**, **d**) of genes in blue module with module membership (MM) > 0.7. GO enrichment analyses (**e**) and PPI network analysis (**f**) of genes in turquoise module with MM > 0.7. GO enrichment analyses of genes in skyblue module, with MM > 0.7 (**g**) Bars representing GO terms show Benjamini and Hochberg FDR adjusted p values. Node size in PPI networks indicates number of interactions with other nodes in the network (i.e., degree centrality), while the node color reflects number of shortest paths that rely on that given node within the network (i.e., betweenness centrality). *dul* duloxetine, *Rum R. flavefaciens*

WGCNA clustering of our RNA-seq data identified a total of 18 modules of co-expressed genes. Module-trait relationship revealed that there were three modules significantly correlated with *R. flavefaciens* treatment that were not correlated with the same directionality with duloxetine treatment (Fig. 5a). The blue module (2904 genes), was positively correlated to the bacterial treatment, while the turquoise module (3353 genes), as well as a small skyblue module (186 genes), were negatively correlated to the bacterial treatment (Fig. 5a). Further confirmations of *R. flavefaciens* effects were strong correlations of module membership (MM) with gene-trait relationship for all three modules (Supplemental Figure S6). In other words, the genes that were strongly correlated to the module eigengenes (i.e., genes with high MM) were also strongly correlated with presence/absence of *R. flavefaciens* treatment.

In order to understand biological mechanisms associated with genes in these three modules, we performed GO and protein-protein interaction (PPI) network analyses on module genes (MM > 0.7). GO analyses of genes in the blue module, which was positively correlated with *R. flavefaciens* treatment, determined enrichment of mitochondrial energy generation processes (Fig. 5b). Subsequent PPI network analyses of the blue module revealed two protein clusters of strongly interconnected gene products also involved in mitochondrial functions. One protein cluster (composed of 32 nodes) mainly consisted of subunits belonging to mitochondrial complex I (NADH:ubiquinone oxidoreductase (NDUF)), while 4 genes are belonging to mitochondrial complex 3 (ubiquinol-cytochrome c reductase complex subunits (UQCR)) (Fig. 5c). The members of the other protein cluster (composed of 14 nodes) are part of mitochondrial ribosomal protein (MRPL) gene family, involved in translation of mitochondrial genes, that are subunits of respiratory chain complex (Fig. 5d). Therefore, *R. flavefaciens* treatment was associated with the upregulation of several mitochondrial pathways.

Turquoise and skyblue modules, whose expression levels were negatively related to *R. flavefaciens* treatment, were both enriched with genes involved in neural functions (Fig. 5e, g). PPI analysis of turquoise module revealed one cluster of 51 proteins, consisting of several smaller highly interacting clusters (Fig. 5f). Gene products of these protein clusters are involved in ionotropic glutamate neurotransmission (such as *Gria1* and *Gria2*, AMPA receptor subunits, and *Grin2a* and *Grin2b*, NMDA receptor subunits), protein phosphorylation (such as *Prkacb*, a subunit of protein kinase A (PKA)), phosphatidylinositol signaling (such as *Pten*) and vesicle-mediated trafficking (such as *Dync1h1* and *Uso1*). Genes from the smallest skyblue module did not give any relevant protein network. Therefore, the WGCNA provided further evidence that *R. flavefaciens* treatment affects gene expression pathways involved in neuronal function, and further highlight the glutamatergic system.

Modules affected by duloxetine treatment (Fig. 5a) were enriched for genes involved in RNA splicing (paleturquise module), transcriptional regulation and MAPK activity (lightgreen module) and protein ubiquitination and cellular localization (tan module) (Supplemental Figure S7), while cyan and darkgrey module did not show enrichment for any GO terms. Therefore, duloxetine also had interesting effects on gene networks. However, these networks were not affected by *R. flavefaciens*, and do not appear to be the target of *R. flavefaciens* effects.

Together, bioinformatical analyses of mPFC transcriptome showed that *R. flavefaciens* treatment up-regulated genes involved in mitochondrial oxidative phosphorylation, while down-regulating genes involved in neural plasticity. Therefore, these putative pathways may explain the modulatory effects of *R. flavefaciens* on antidepressant actions on depressive-like behavior.

### Effects of *R. flavefaciens* on serotonin and noradrenalin effects in mPFC

To further illuminate the mechanism by with *R. flavefaciens* exerts its behavioral effects, we also evaluated levels of serotonin and noradrenalin in mPFC after chronic duloxetine and/or bacteria treatment (Supplemental Figure S8). Levels of serotonin (Supplemental Figure S8a) and noradrenaline (Supplemental Figure S8a) were not changed after chronic duloxetine administration. However, two-way ANOVA showed overall effect of *R. flavefaciens* treatment on serotonin levels (F_Rum_ = 4.61, *p* < 0.05) (Supplemental Figure S8a), and a tendency of overall bacteria effect on noradrenaline levels (F_Rum_ = 3.85, *p* = 0.06) (Supplemental Figure S8b).

## Discussion

This study demonstrates that different types of commonly used drugs for alleviating depressive symptoms, i.e., serotonin and/or norepinephrine reuptake inhibitors, have common effects on the composition of gut microbiota and that the microbiome may play a role in their antidepressant actions. Despite their widespread use for several decades, revealing mechanisms of SSRI and SNRI actions, and potential causes of individual differences in responsiveness to them, is still a topic of research, being that they are the most prescribed antidepressants, yet with limited therapeutic effects. Our study illuminates one more pathway of their activity, i.e., through changes of the gut microbiota, suggesting an additional mechanism through which we may be able to modulate and improve their therapeutic effects. Likewise, it was recently shown that some other psychotropic drugs can modulate gut microbiota, and these changes were related to their metabolic side effects or antidepressant effects^57,58^.

Regarding the antidepressant effects on overall gut bacterial diversity, all the drugs except desipramine reduced richness of mice gut microbiota, while simultaneously increasing beta diversity. Such changes in microbial diversity are in agreement with the metacommunity theory. According to this theory, reduced alpha diversity of gut microbial communities, resulting in reduced dispersal of symbionts, can lead to bigger differences between local communities, i.e., higher beta diversity^59–61^. It is generally accepted that higher gut bacterial diversity is beneficial for individual health, and decreased microbiota richness is associated with disease states, such as irritable bowel disease^62,63^ or obesity^64^. Therefore, reduced gut bacterial richness after antidepressant treatments could be of potential health concern for causing possible side effects.

The main goal of our gut microbial community analyses was to investigate whether we can identify specific bacterial taxa which change in the same way by all tested antidepressants, presuming that such alterations can underline their common antidepressant effects. Antidepressants reduced relative abundances of three genera: *Ruminococcus*, *Adlercreutzia* and an undefined genus in the order RF32, class Alphaproteobacteria. These changes can be considered to be in accordance with previous *in vitro* studies showing that monoamine reuptake inhibitors can have antimicrobial effects^21,65,66^, although there are no data regarding effects on species we found altered. Indeed, recent screen of more than 1000 commonly used human drugs showed that nearly one fourth of non-antibiotic mediation exhibit anticommensal activity^66^. Although the study revealed that several monoamine reuptake inhibitors inhibit growth of at least one bacterial species, for the antidepressants we also used in the study (fluoxetine, escitaloprame, venlafaxine and desipramine) none of the antimicrobial effects were shown^66^. The reason for these discrepancies could be that the species they used in the screen are not sensitive to these antidepressants and/or that the applied concentrations are below those that are achieved in gut after chronic i.p. treatment with doses of antidepressants we used.

Currently, not much is known about how antidepressants achieve these antimicrobial effects. A few studies suggest they can act as efflux pump inhibitors affecting bacterial quorum sensing^21,22,67^. On the other hand, it is shown that antidepressants can bind to bacterial homologs of Na^+^/Cl^−^-dependent neurotransmitter transporters, a family of proteins to which both serotonin transporter and norepinephrine transporter belong to, and inhibit their activity^68,69^

In the following series of experiments, we examined if decrease of *Ruminococcus* and/or *Adlercreutzia* by antidepressant drugs is causally related to their antidepressant effects. Indeed, in mice treated by duloxetine while concurrently supplemented with *R. flavefaciens*, antidepressive properties of duloxetine were attenuated. This effect of *R. flavefaciens* was evident in tests assessing rodent behavioral despair (i.e., TST and FST), as well as anhedonia. Supplementation with *A. equolifaciens* did not abolish duloxetine antidepressive effects, therefore demonstrating the specificity of the effects for *R. flavefaciens*. Involvement of *Ruminococcus* species in depressive behavior was also suggested by some other recent studies. Namely, in a study exploring involvement of gut microbiota in antidepressant properties of docosahexaenoic acid (DHA), positive correlation was found between abundance of *Ruminococcus* and anhedonic behavior of mice^70^. Further, prebiotics which exhibited antidepressive effects, also reduced abundance of the genus *Ruminococcus*^71^. In the current study, we directly demonstrate that introduction of a single *Ruminococcus* species, i.e., *R. flavefaciens*, is able to abolish antidepressive effects of an antidepressant. This confirms that changes in gut microbiota can be causally related to antidepressant properties of antidepressant drugs.

It is interesting that, contrary to undesirable effects of *R. flavefaciens* regarding antidepressant effects of duloxetine, replenishing of this bacteria was shown to be beneficial regarding antidepressant-induced constipation. Namely, duloxetine, as other antidepressants, are known to produce constipation as side-effect in certain individuals^72,73^. However, *R. flavefaciens* treatment was able to abolish this. To our best knowledge, there is no data about how *R. flavefaciens* could achieve this effect, but we can speculate it could include its involvement in degradation of complex carbohydrates and production of short-chain fatty acids^74–76^. However, reduced defecation caused by duloxetine in open field could be interpreted as an anxiolytic effect, and not necessarily related to constipation. We do note that we did not observe any differences in time spent in the center of the open field by any of treatments (data not shown), which is also a sign of anxiety behavior^54^. Therefore, the effect of treatment by *R. flavefaciens* on the defecation should be interpreted with caution, since defecation can be effected by both gut abnormalities (constipation) and anxiety-like behavior.

Whole transcriptome analysis of mPFC revealed that *R. flavefaciens* treatment induced an increase of genes involved in mitochondrial oxidative phosphorylation, specially pointing to subunits of complex I and MRPLs. Impairment of mitochondrial electron transport chain has already been implicated in etiology of mood disorders, although the evidence regarding the directionality of expression changes are mixed^77–79^. Also, it is shown that antidepressants can influence mitochondrial electron transport chain activity, and although their effects are still not clear, they suggest their importance for antidepressant therapeutical actions^80,81^. A potential mechanism how *R. flavefaciens* could affect mitochondrial energy metabolism is by affecting the fermentation of carbohydrates and subsequent production of short-chain fatty acids, that were shown to interfere with oxidative phosphorylation^76,82,83^.

Further, *R. flavefaciens* down-regulated genes involved in synaptic signaling and neurogenesis, including genes encoding ionotropic glutamate receptor subunits and a PKA subunit. Indeed, impaired neuroplasticity in depression is well documented, and antidepressant treatment can often rescue deficits in neuroplasticity^17,84^. Specifically, there are accumulating evidences indicating the importance of glutamatergic synapse dysfunction in etiology of depression, including decreased expression and binding of AMPA and NMDA receptor subunits^85–87^. Additionally, regulation of glutamate receptor function have been shown to be implicated antidepressant effects as well^88^. Likewise, diminished expression of PKA subunits, and PKA activity, has been reported in patients with depression, while efficient antidepressant treatment has been related to increased PKA activity^89–91^.

There may be a connection between *R. flavefaciens* effects on mitochondrial processes and synaptic signaling. Namely, ATP is fundamental for numerous neuronal process, involved in proper synaptic functioning and plasticity^92,93^. However, electron transport chain is also a main source of reactive oxygen species generation that could be detrimental for neurons^94,95^. This may explain the association between increased expression of mitochondrial related genes and decreased neuroplasticity genes in animals treated with *R. flavefaciens*.

It was surprising that DEA identified only one gene affected by duloxetine treatment, namely *Adrb1* norepinephrine receptor. However, WGCNA revealed several networks, already well-known to be affected by antidepressant treatments, such as glucocorticoid receptor and MAPK signaling^89,96–98^, as well as mRNA processing and protein ubiquitination.

Duloxetine and *R. flavefaciens* exhibited different effects on gene transcription networks in the brain. This is not surprising, considering that antidepressants produce a wide spectrum of actions directly in brain, apart from their activity on the microbiota. However, it is plausible that the gene expression changes in synaptic and mitochondrial genes, induced by *R. flavefaciens*, may contribute to attenuation of the antidepressant properties by modifying neuronal functions, and making them less reactive to the properties of antidepressants. Therefore, dysregulated mitochondrial function, along with decreased neuroplasticity in mPFC could contribute to *R. flavefaciens* attenuation of antidepressant effects on depressive-like behavior.

Finally, we showed that *R. flavefaciens* treatment reduced levels of serotonin and noradrenaline in mPFC, which could mediate abolishment of antidepressive effects by the bacteria and observed changes in transcriptome. Although we did not find any changes of serotonin and noradrenaline after duloxetine treatment, it could be that these changes were transient, and at the end of chronic treatment (25-26^th^ day) they could not been observed anymore^99–101^. In addition, it is possible that duloxetine only affected levels of these neurotransmitters in the synaptic cleft, but not total levels in the tissue, which we examined. While the mechanism of the *R. flavefaciens* effect is not clear, this finding suggests that modulation of monoamine neurotransmitters may partly explain the effect of *R. flavefaciens* on depressive-like behavior.

There are several limitations in this study which should be kept in mind to properly interpret their results, as well as to plan for future studies. First, this study was only performed on male mice, and it would be of further interest to expand these studies in female mice. Secondly, the animals used in this study were not subjected to a stress protocol. While the BALB/C model is used as a depressive-like mouse model, which responds to antidepressant treatment, it is true that depression is often triggered by trauma or stressful events, which were not imitated in this current study. Third, it is also important to point out that the observed microbial changes are attained after chronic treatment with antidepressants (21 day treatment). While in humans an approximately 21 day antidepressant treatment is usually needed before seeing behavioral effects, acute treatment in mice is adequate to see immediate behavioral effects. Therefore, acute effects of antidepressants in mice are likely not due to the microbial changes we have observed. Further studies would be necessary to understand the effect of microbiome on the acute effects of antidepressants on behavior in mice.

Although at the present moment, it seems harder to therapeutically achieve bacterial depletion, than to replenish them, it is likely that improved understanding of factors that reduce or stimulate growth of certain bacteria can give promise as an option to enhance antidepressant therapeutical effects through modulating gut microbiota. For example, prebiotics, as fructo-oligosaccharides and galacto-oligosaccharides, decrease *Ruminococcus*, among other gut bacterial changes, and therefore their joined administration with antidepressants to resistant individuals could provide a potential way to achieve favorable gut microbiota balance that would lead to alleviation of depression.

In conclusion, our results provide evidence for antidepressant effects on gut microbiota. Specially, the study reveals the importance of decreasing *R. flavefaciens* for antidepressants to achieve their therapeutical effects, i.e., to reduce depressive behavior. The mechanism of these bacterial actions may involve impairment of mitochondrial oxidative phosphorylation and neural plasticity in mPFC.

## Supplementary information

Supplementary Figures and Methods
