# Supplementary material for: Antidepressants affect gut microbiota and Ruminococcus flavefaciens is able to abolish their effects on depressive-like behavior
Source: Transl Psychiatry. 2019 Apr 9;9:133. doi: 10.1038/s41398-019-0466-x (PMC6456569; doi:10.1038/s41398-019-0466-x)
Supplement: Supplementary file 1 — Supplementary Figures and Methods [file 41398_2019_466_MOESM1_ESM.doc]

**Antidepressants affect gut microbiota and *Ruminococcus flavefaciens* is able to abolish their effects on depressive-like behavior**

Supplemental Information

**Supplemental methods and materials**

**Behavioral testing**

***Tail suspension test (TST).*** Mice were suspended by their tail for six minutes, with the tape applied about 1cm distance from the tip of their tail, under low light conditions (25 lux). Behavior other than vigorous trying to escape was scored as immobility, and considered as indicator of depressive-like behavior (small movements with only front legs as well as swinging were not considered as mobility) ^1^. The maze was cleaned with 10% alcohol after testing of each mouse. All testing was recorded by a video camera, and scoring was done manually.

***Forced swim test (FST).*** We used modified forced swim test procedure with pre-test 24 h before the test day ^2^. Both pre-test and test lasted 6 minutes and were conducted in plastic buckets 21 cm in diameter and filled with 24±1^o^C water 15 cm from the bottom, under low light conditions (25 lux). After testing, mice were dried, and then returned to their home cages. Immobility, defined as absence of active swimming with all four paws and tail moving, was scored during all six minutes on the test day as sign of depression-like behavior ^2,3^. All experimentation was recorded by a video camera, and scoring was done manually. The test was preformed two days after tail suspension test.

***Sucrose preference test (SPT).*** Sucrose preference test was used to assess anhedonia in mice. During this test, mice were single-housed, and presented with 2 pippets to choose freely: one containing drinking water and the other one 2% sucrose solution. The position of pippets was switched every 12 hours to eliminate side preference as a confounder. The test lasted two days, with first 24 hours considered as habituation, while the next 24 hours were considered as test day. During that time consumption of water and sucrose solution was measured and sucrose preference was calculated as a percentage of the volume of sucrose solution intake divided by the volume of total fluid intake. Sucrose preference was performed six days after forced swim tests.

***Open field test.*** A mouse was gently placed in the corner of the arena (50 x 50 cm, illuminated by 120 lux) and recorded during 10 min by video-computerized tracking system Ethovision. The total distance traveled was used as an index of locomotor activity. Also, faecal output was recorded during the test, as a number of produced stool pellets at the end of the test ^4,5^.

***Rotarod.*** Mice were placed at on rotating rods of rotarod apparatus, facing away from direction of rotation. Rotarod started with a speed of 4 rpm, and accelerated to 40 rpm in 5 min time. The time at which mouse fell was recorded and expressed as percentage of maximum time (5 min). All mice preformed rotarod test twice.

**Stool collection, DNA extraction and sequencing of 16s rRNA gene**

After DNA isolation, the V4 region of bacterial 16S rRNA gene was PCR-amplified using the 515F and 806R primers^6^. Forward primers included unique 12-base barcodes in order to tag PCR products from different samples. PCR reaction consisted of PrimeSTAR Max Premix 1x (Takara Bio), 0.4 μM of each primer and 30-100 ng DNA template. Reaction conditions were as following: initial denaturing step for 3 min at 95^o^C, followed by 30 cycles of 10 s at 95^o^C, 5 s at 55^o^C and 5 s at 72^o^C. PCR reactions were performed in duplicates for each sample, pooled and purified with Agencourt AMPure XP kit (Beckman Coulter). Purified PCR products were quantified using a Qubit dsDNA HS assay kit (Life Technologies) and 50 ng of each sample was pooled for further sequencing on the Illumina MiSeq platform.

**Bioinformatic analyses of 16S rRNA gene sequences**

The data from 16s rRNA sequencing were analyzed by QIIME 1 pipeline ^7^. After joining reverse and forward reads, quality filtering and chimera checking (by USEARCH61) ^8^, open-reference picking operational taxonomic units (OTUs) was done at 97% similarity, followed by taxonomical classification using the Greengenes reference database ^9^. OTUs that were not present in at least one group in more than 50% of samples were filtered out. To normalize sequence counts across samples, 41.600 sequences were randomly selected per sample (rarefaction) for diversity analyses and for comparing abundances of OTUs across samples. For phylogenetic tree-based analyses, representative sequences of each OTU were aligned using PyNAST ^10^ and a phylogenetic tree was constructed using FastTree ^11^.

**PCR conformation of the bacteria in the stool**

The presence of *R. flavefaciens* 17 and *A. equolifaciens* FJC-B9 in the stool samples of treated mice was confirmed by PCR. PCR was performed as described in the methods section in the manuscript, using PrimeSTAR Max Premix 1x, 0.4 μM of appropriate primers and 10-100 ng of template DNA, and the same PCR reaction conditions stated in the main manuscript. The primer sequences can be found in Supplementary table S1. The products were visualized using 2% agarose gel electrophoresis and ethidium bromide.

**Bioinformatic analyses of mRNA sequencing**

After mRNA sequencing, gene count normalization (10 million sequencies per sample) as well as differential expression analysis were done using 'DESeq2' R package. Significant differential expression was considered according to a threshold of 0.05 on Benjamini and Hochberg FDR adjusted *p* values.

In weighted gene correlation network analysis (WGCNA)^12^, Pearosn's correlations between gene expression data were used to build a signed network. A soft threshold of 7 was used for creating the adjacency matrix, chosen according to scale-free topology criterion. Next, Topological Overlap Matrix (TOM)-based dissimilarity measure was used for constructing dendogram of the network, and single modules, corresponding to dendogram branches, were defined using Dynamic Tree Cut algorithm. All modules, that essentially represent clusters of highly interconnected genes, were given a color. The module eigengene (ME) was calculated for every sample in each module. ME values correspond to the first principal component of the particular module and can be considered as a representative of expression profiles of genes from that module. To evaluate module-trait associations, the ME values of samples from each module were correlated to applied treatments. Also, WGCNA defines the module membership (MM) for each gene, which represents the correlation between its expression levels and the ME values across all samples. For those modules that showed significant correlation with *R. flavefaciens* treatment, additionally, we evaluated association between MM and gene-trait relationship. In our case, gene-trait relationship represented the correlation between the expression levels of genes from the particular module with presence/absence of *R. flavefaciens* treatment.

For protein-protein interaction (PPI) network analysis, the desired sets of genes were firstly loaded into the STRING database, where we searched for high confidence protein interactions (0.7). Further network analyses were performed with Cytoscape (version 3.2.1). A network topology was based on the confidence scores calculated by STRING. After obtaining an initial network of desired set of genes, we used the Cytoscape MCODE v1.5 application to locate protein clusters of highly interconnected regions within the initial network that belongs to the same biological pathway. Network characteristics such as degree centrality (i.e. measure of node interactions with other nodes in the network) and betweenness centrality (i.e. measure of shortest paths that rely on that given node within the network) were obtained and used to locate important nodes in the network.

**Supplemental figures**

**
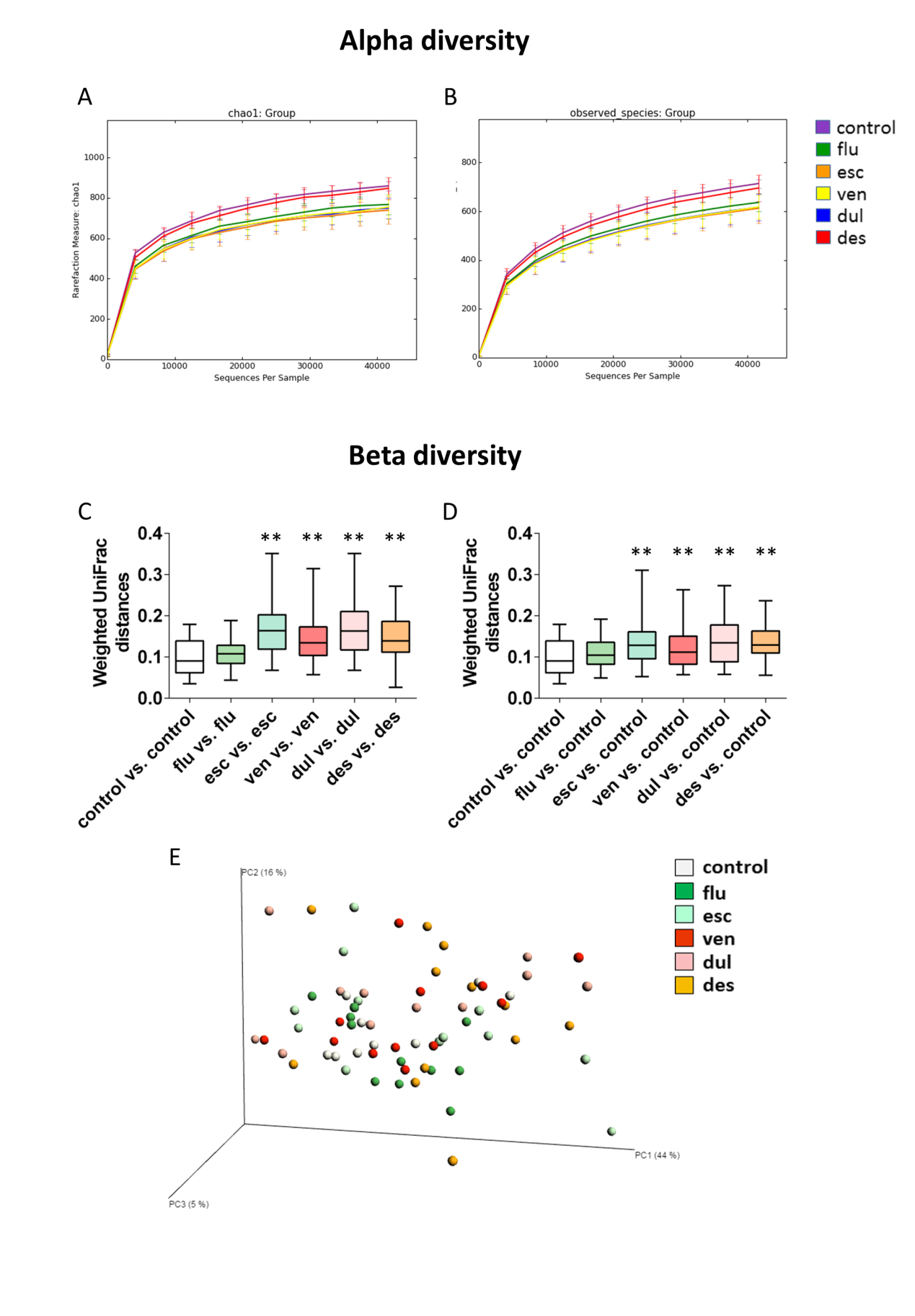
**

**Figure S1.**

**(A, B)** Measures of alpha diversity. Chao1 (A) and number of observed species (B) rarefaction curves representing the reduction in richness of gut microbiota in all antidepressant treated mice except desipramine, relative to controls.

**)C-E)** Measures of beta diversity estimated by Weighted UniFrac distances of microbial communities of antidepressant treated mice in comparison to control samples. Weighted UniFrac-based PCoA plot that visualizes microbial communities of all antidepressant treated and control mice (the percentage of variation explained by the principal coordinates is indicated on the axes) (E). ** p<0.01, FDR corrected, nonparametric t-tests with 999 Monte Carlo permutatins, in comparison to control group; n=9 (control), n=11 (flu), n=12 (esc), n=12 (ven), n=11 (dul), n=12 (des), animals per group; data represent mean ± SEM.

Abbreviations: fluoxetine (flu), escitalopram (esc), venlafaxine (ven), duloxetine (dul) and desipramine (des).


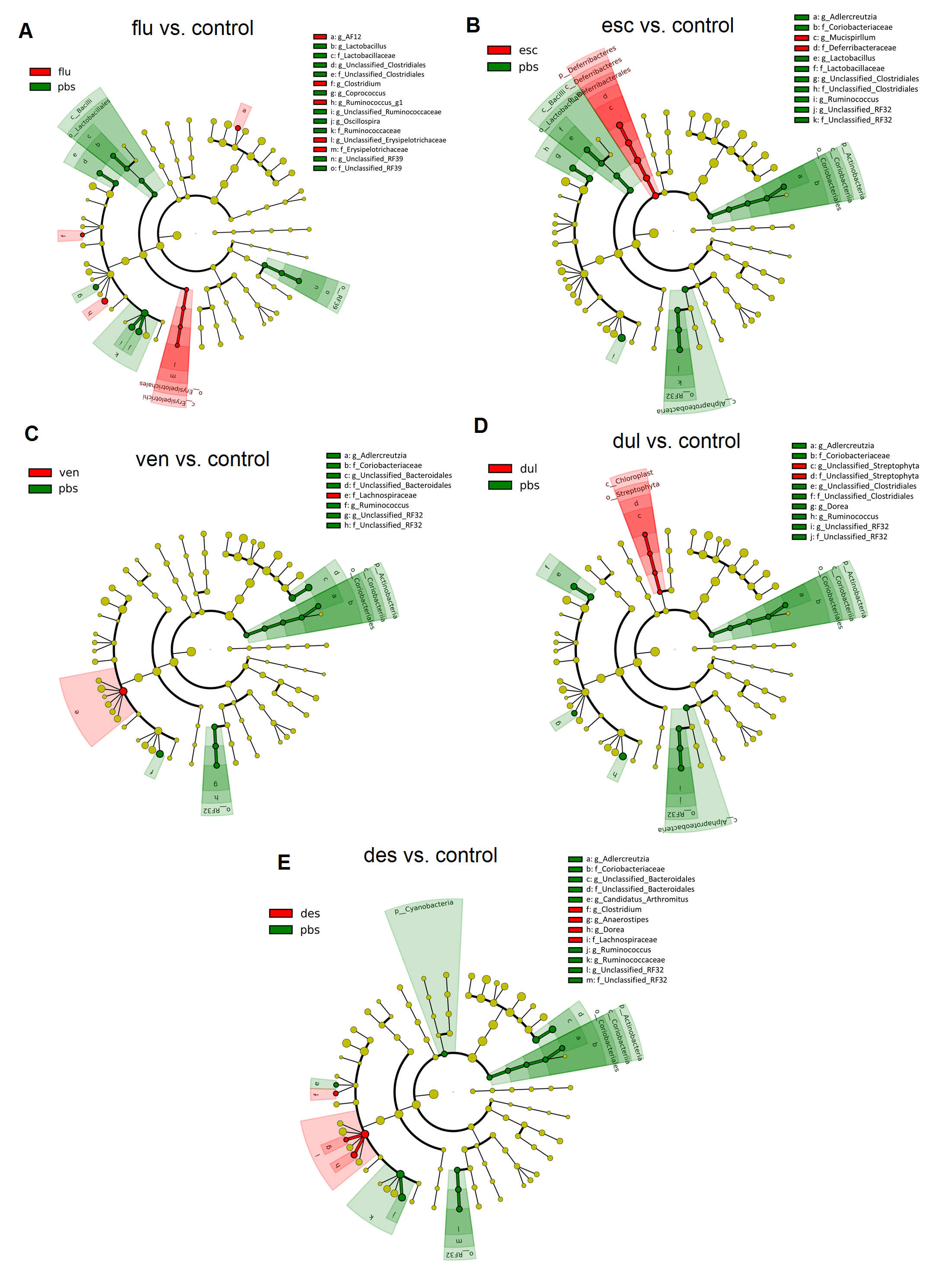


**Figure S2**.

Taxonomic cladograms represent bacterial taxa differently abundant in stool samples of each of antidepressant treated groups compared to controls in pairwise fashion.

Cladograms show differences between control and fluoxetine (A), control and escitalopram (B), control and venlafaxine (C), control and duloxetine (D), and control and desipramine (E). RED - bacterial taxa more abundant in antidepressant treated mice, and GREEN - bacterial taxa more abundant in control mice (LEfSe, p<0.05, LDA>2). *Ruminococcus* and *Adlercreutzia*, among others, were decreased in pairwise tests between control and escitalopram (B), venlafaxine (C), duloxetine (D), and desipramine (E). n=9 (control), n=11 (flu), n=12 (esc), n=12 (ven), n=11 (dul), n=12 (des), animals per group.

Abbreviations: fluoxetine (flu), escitalopram (esc), venlafaxine (ven), duloxetine (dul) and desipramine (des).


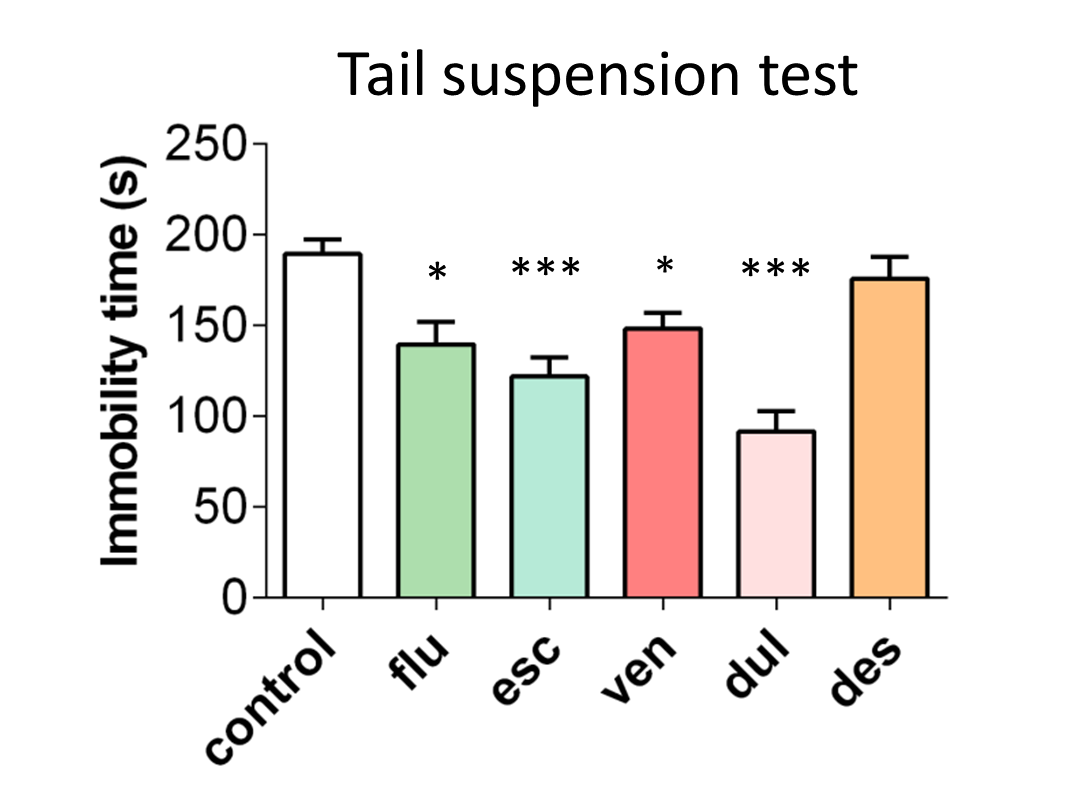


**Figure S3.**

All antidepressants, except desipramine, reduced depressive-like behavior in tail suspension test (one-way ANOVA: F=11.70, p<0.001).

* p<0.05, ** p<0.01, *** p<0.001, Dunnett’s post hoc test, in comparison to control group; n=14 (control), n=11 (flu), n=12 (esc), n=12 (ven), n=12 (dul), n=12 (des), animals per group; data represent mean ± SEM.

Abbreviations: fluoxetine (flu), escitalopram (esc), venlafaxine (ven), duloxetine (dul) and desipramine (des).


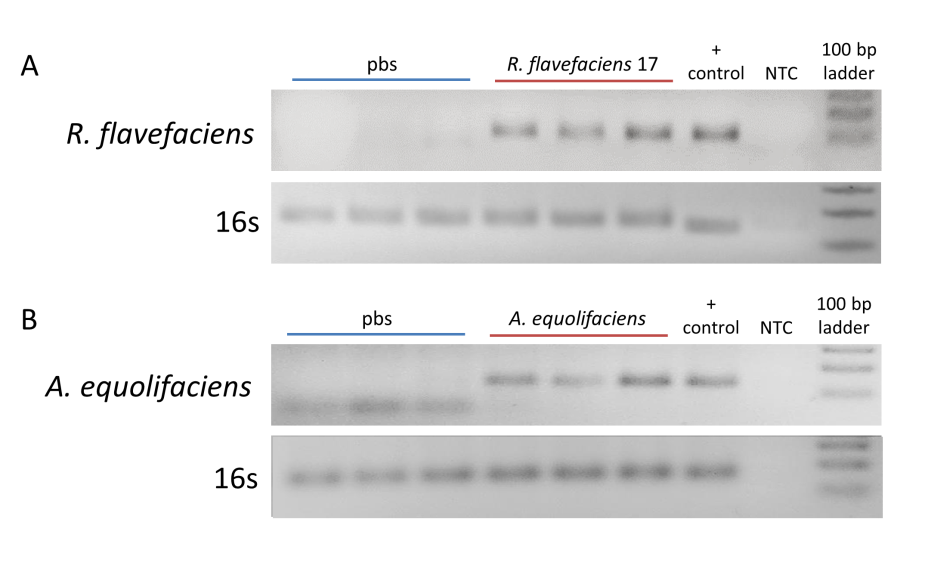


**Figure S4.** The presents of *R. flavefaciens* 17 and *A. equolifaciens* FJC-B9 in the feces of treated mice confirmed by PCR and 2% agarose gel electrophoresis.

**(A, B)** Upper pictures show PCR products specific for the bacteria given by gavage (*R. flavefaciens* 17 (A) or *A. equolifaciens* FJC-B9 (B)), while down pictures show PCR products of 16s RNA gene, as positive control for each of the samples. The samples on the gels are at the following order: 3 control samples gavaged with pbs, 3 samples gavaged with bacteria (*R. flavefaciens* 17 (A) or *A. equolifaciens* FJC-B9 (B)), sample of corresponding bacterial culture as a positive control (+ control), and sample of no template control (NTC). n = 3 randomly chosen animals per group.

**
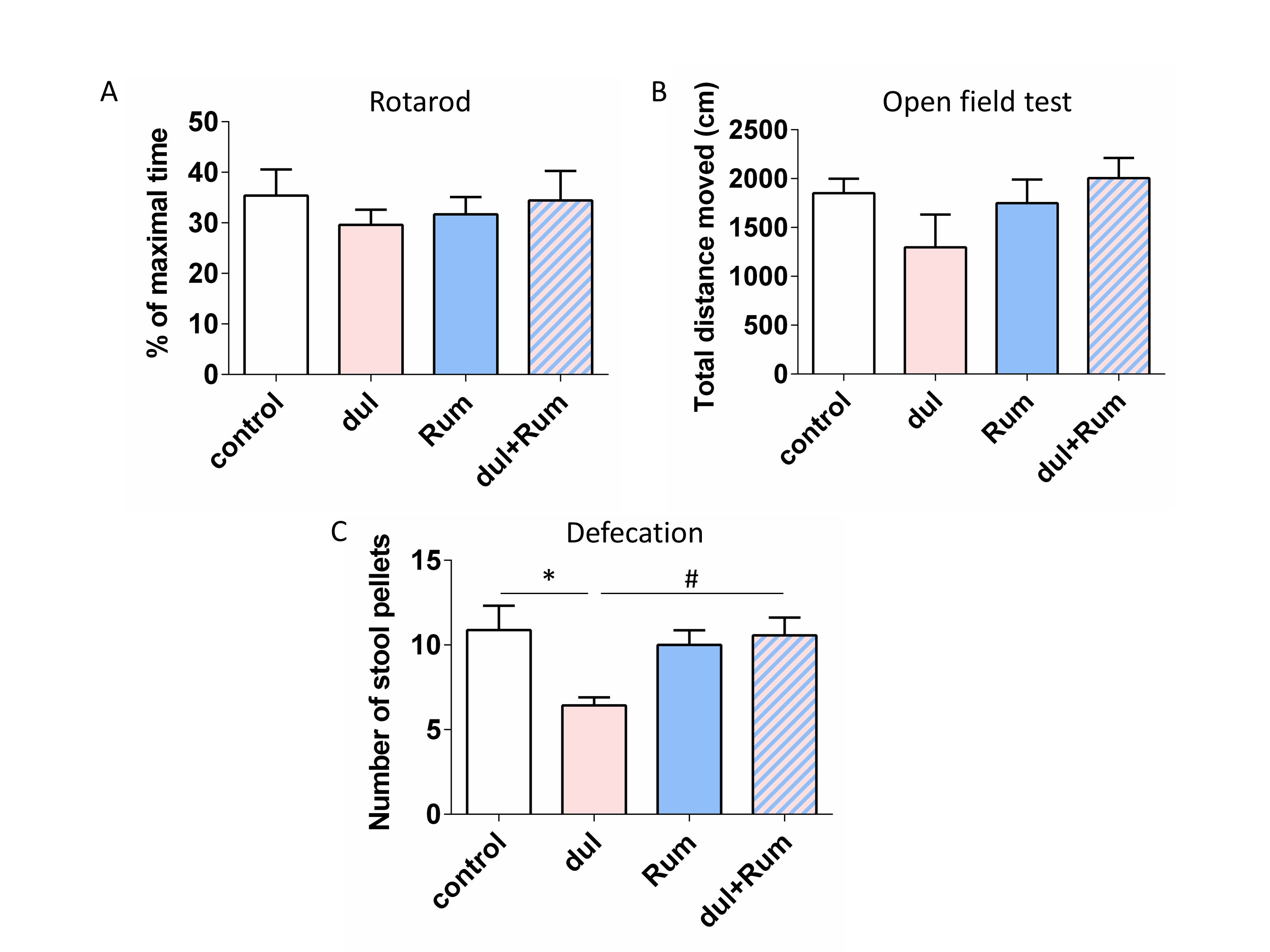
Figure S5**. Effects of duloxetine and *R.flavefaciens* on locomotion and gastrointestinal health.

(A, B) Locomotion was evaluated by time to fall in rotarod test and total distance moved in open field test. In open field test, two-way ANOVA showed effect of *R.flavefaciens* (F=5.41, p<0.05) and effect of duloxetine and *R.flavefaciens* interaction (F=8.13, p<0.01). No differences were detected between groups.

(C) Defecation was evaluated during open field test. Two-way ANOVA showed tendency of duloxetine effect (F=3.4, p=0.08) and effect of duloxetine and *R.flavefaciens* interaction (F=5.70, p<0.05).

n=8 (control), n=8 (dul), n=8 Rum), n=7 (dul+Rum), animals per group.

# 1>p>0.05, * p<0.05, post hoc Tukey’s test; data are represented as mean ± SEM.

Abbreviations: duloxetine (dul) and *R. flavefaciens* (Rum).

**
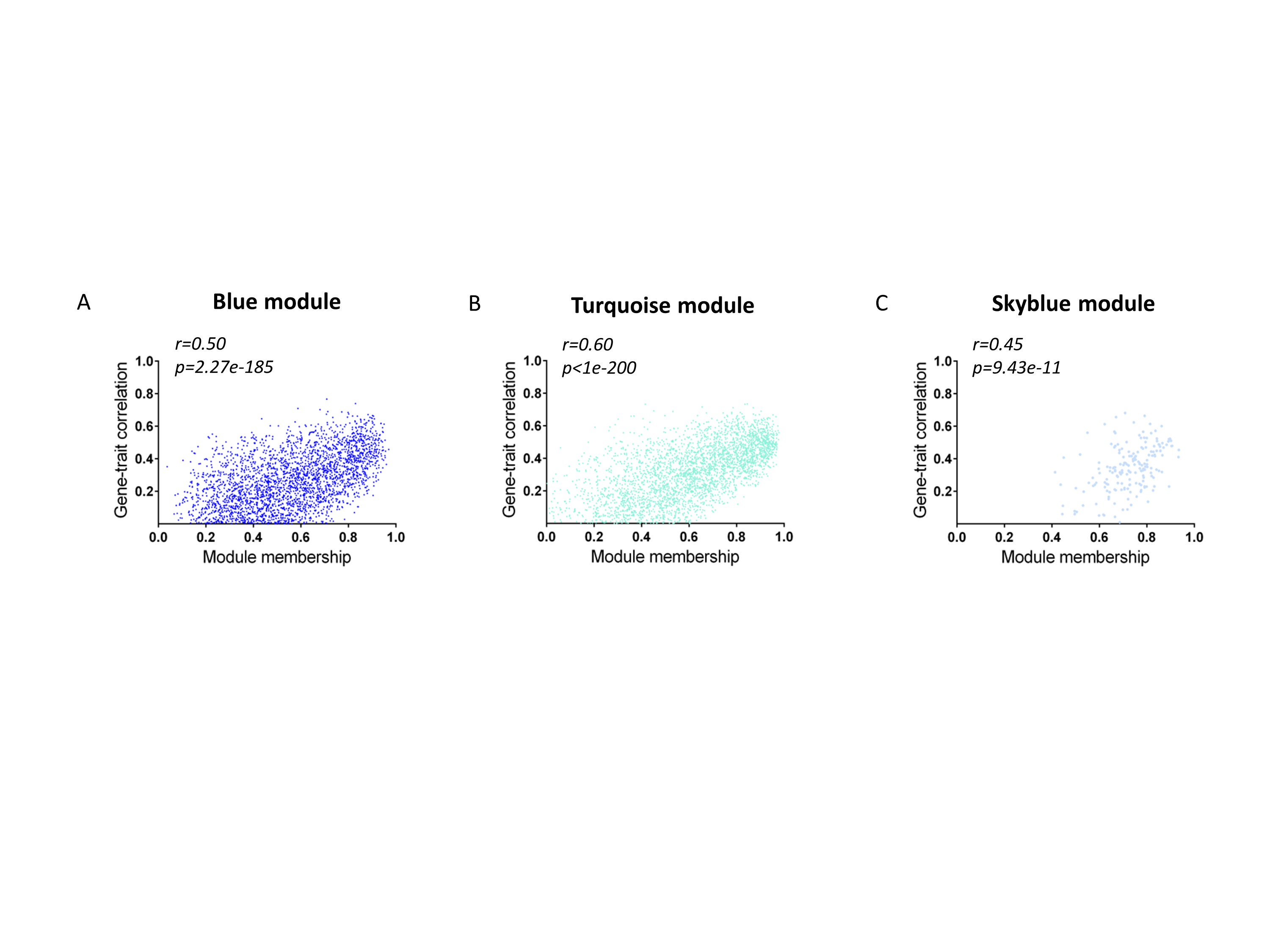
**

**Figure S6.** Scatter plots represent gene-trait correlations with module membership (MM) for blue (A), turquoise (B) and skyblue (C) modules, as conformation of *R. flavefaciens* effects. Correlation coefficients (Pearson’s r correlation) and corresponding *p* values are indicated on the each plot.


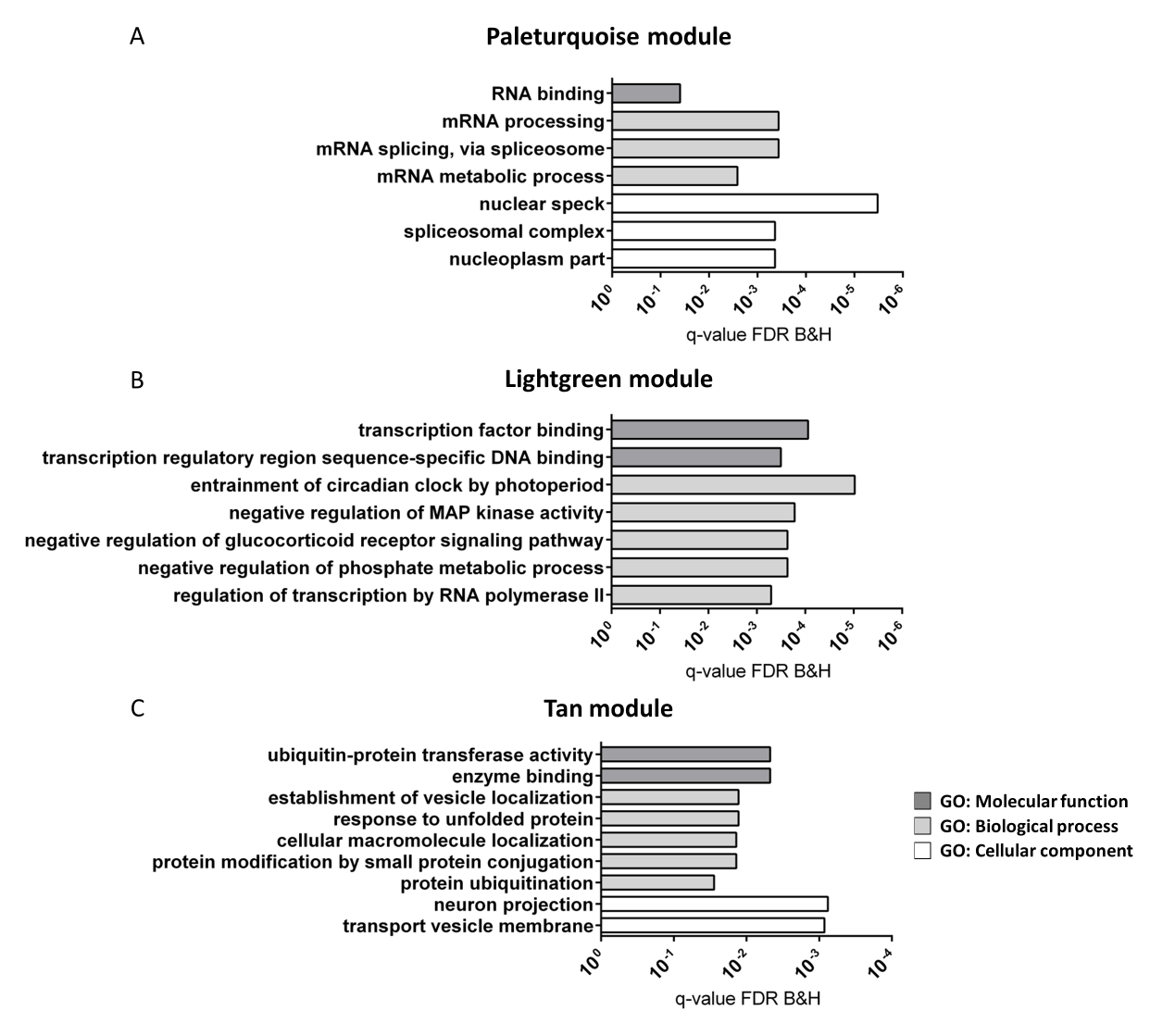


**Figure S7.** Gene ontology (GO) analysis of WGCNA modules significantly related to duloxetine treatment.

)A) GO enrichment analyses of genes in paleturquoise module with module membership (MM) > 0.7.

)B) GO enrichment analyses of genes in lightgreen module with MM > 0.7.

)C) GO enrichment analyses of genes in tan module, with MM > 0.7.

Bars representing GO terms show Benjamini and Hochberg FDR adjusted *p* values.

**
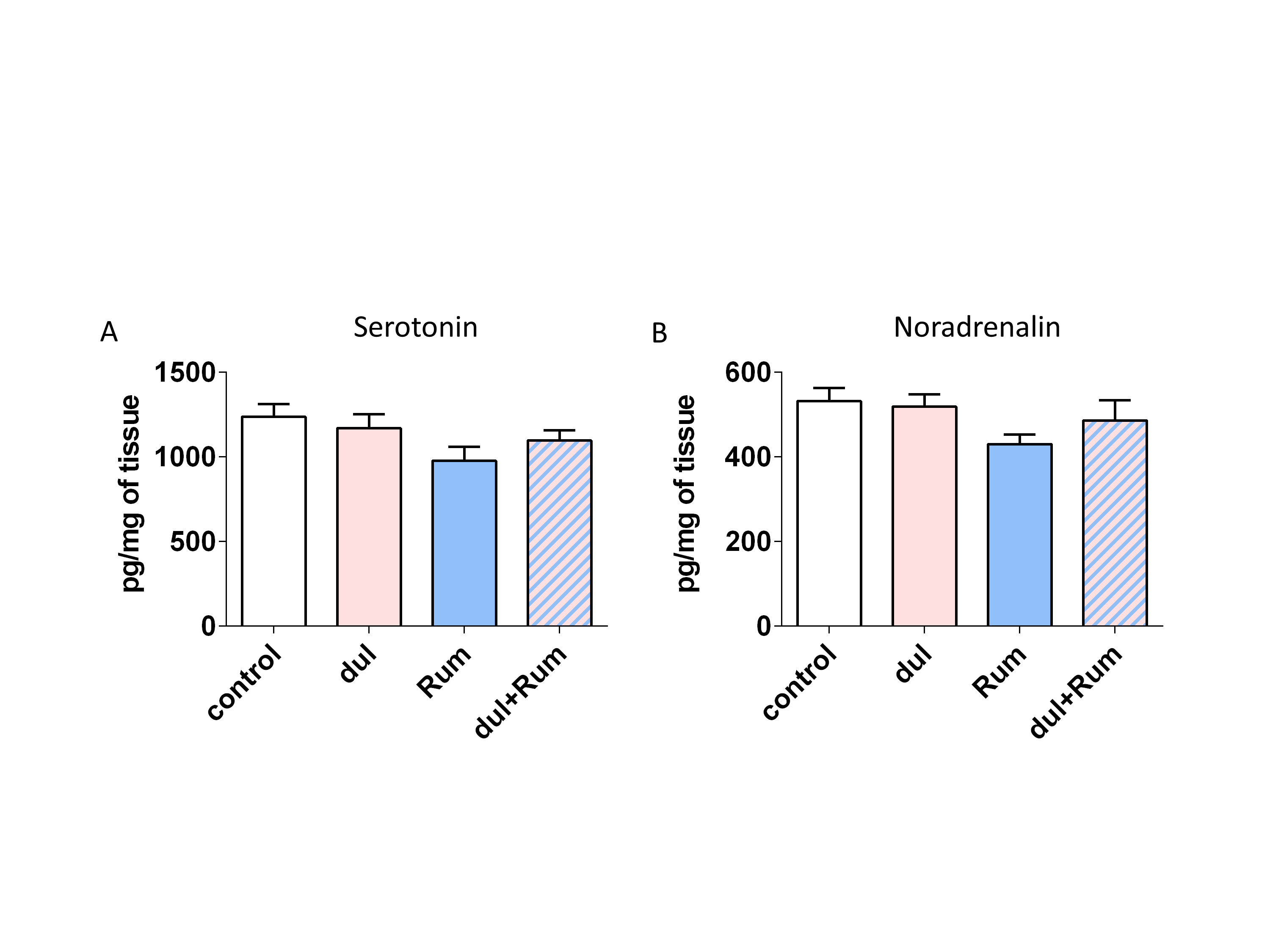
Figure S8**. Effects of duloxetine and *R.flavefaciens* on serotonin and noradrenalin levels in mPFC.

(A) *R. flavefaciens* treatment reduced serotonin levels (two-way ANOVA: F_Rum_ = 4.61, p<0.05).

(B) *R. flavefaciens* treatment showed tendency for reduction of noradrenalin levels, (two-way ANOVA: F_Rum_=3.85, p=0.06).

Data are expressed as picograms of neurotransmitters per mg of tissue. n=8 (control), n=8 (dul), n=8 Rum), n=7 (dul+Rum), animals per group. p=0.1, post hoc Tukey’s test; data are represented as mean ± SEM.

Abbreviations: duloxetine (dul) and *R. flavefaciens* (Rum).

**Supplementary tables**


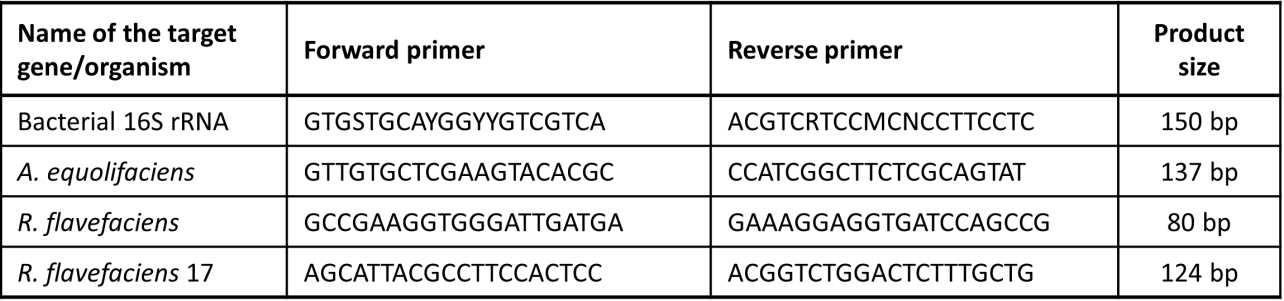


**Table S1.** Sequences of primers used in the study.

**REFERENCES:**

1 Can A, Dao DT, Terrillion CE, Piantadosi SC, Bhat S, Gould TD. The Tail Suspension Test. *J Vis Exp* 2011; : e3769–e3769.

2 Dulawa SC, Holick KA, Gundersen B, Hen R. Effects of Chronic Fluoxetine in Animal Models of Anxiety and Depression. *Neuropsychopharmacology* 2004; **29**: 1321–1330.

3 Can A, Dao DT, Arad M, Terrillion CE, Piantadosi SC, Gould TD. The Mouse Forced Swim Test. *J Vis Exp* 2011; : e3638–e3638.

4 Baek I-S, Park J-Y, Han P-L. Chronic Antidepressant Treatment in Normal Mice Induces Anxiety and Impairs Stress-coping Ability. *Exp Neurobiol* 2015; **24**: 156.

5 Davey KJ, Cotter PD, O’Sullivan O, Crispie F, Dinan TG, Cryan JF *et al.* Antipsychotics and the gut microbiome: olanzapine-induced metabolic dysfunction is attenuated by antibiotic administration in the rat. *Transl Psychiatry* 2013; **3**: e309.

6 http://www.earthmicrobiome.org/.

7 Caporaso JG, Kuczynski J, Stombaugh J, Bittinger K, Bushman FD, Costello EK *et al.* QIIME allows analysis of high-throughput community sequencing data. *Nat Methods* 2010; **7**: 335–336.

8 Edgar RC, Haas BJ, Clemente JC, Quince C, Knight R. UCHIME improves sensitivity and speed of chimera detection. *Bioinformatics* 2011; **27**: 2194–2200.

9 McDonald D, Price MN, Goodrich J, Nawrocki EP, DeSantis TZ, Probst A *et al.* An improved Greengenes taxonomy with explicit ranks for ecological and evolutionary analyses of bacteria and archaea. *ISME J* 2012; **6**: 610–8.

10 Caporaso JG, Bittinger K, Bushman FD, DeSantis TZ, Andersen GL, Knight R. PyNAST: a flexible tool for aligning sequences to a template alignment. *Bioinformatics* 2010; **26**: 266–267.

11 Price MN, Dehal PS, Arkin AP. FastTree: Computing Large Minimum Evolution Trees with Profiles instead of a Distance Matrix. *Mol Biol Evol* 2009; **26**: 1641–1650.

12 Langfelder P, Horvath S. WGCNA: an R package for weighted correlation network analysis. *BMC Bioinformatics* 2008; **9**: 559.
